# Sex differences in the cardiac stress response following SARS-CoV-2 infection of ferrets

**DOI:** 10.1152/ajpheart.00101.2023

**Published:** 2023-09-22

**Authors:** Sarah Rouhana, Kathy Jacyniak, Magen E. Francis, Darryl Falzarano, Alyson A. Kelvin, W. Glen Pyle

**Affiliations:** ^1^IMPART Investigator Team, Dalhousie Medicine, Saint John, New Brunswick, Canada; ^2^Department of Biomedical Sciences, University of Guelph, Guelph, Ontario, Canada; ^3^Department of Biochemistry, Microbiology, and Immunology, University of Saskatchewan, Saskatoon, Saskatchewan, Canada; ^4^Vaccine and Infectious Disease Organization-International Vaccine Centre, University of Saskatchewan, Saskatoon, Saskatchewan, Canada

**Keywords:** COVID-19, heart, myocardial stress, SARS-CoV-2, sex differences

## Abstract

Severe acute respiratory syndrome coronavirus 2 (SARS-CoV-2) infection damages the heart, increasing the risk of adverse cardiovascular events. Female sex protects against complications of infection; females are less likely to experience severe illness or death, although their risk for postacute sequelae of COVID-19 (“long COVID”) is higher than in males. Despite the important role of the heart in COVID-19 outcomes, molecular elements in the heart impacted by SARS-CoV-2 are poorly understood. Similarly, the role sex has on the myocardial effects of SARS-CoV-2 infection has not been investigated at a molecular level. We intranasally inoculated female and male ferrets with SARS-CoV-2 and assessed myocardial stress signals, inflammation, and the innate immune response for 14 days. Myocardial phosphorylated GSK3α/β decreased at *day 2* postinfection (pi) in male ferrets, whereas females showed no changes. Myocardial levels of p62/SQSTM1 decreased in male ferrets at *days 2*, *7*, and *14* pi while lower baseline levels in females increased on *day 2*. Phosphorylated ERK1/2 increased in cardiomyocyte nuclei in females on *days 2* and *14* pi, whereas male ferrets had no changes. Only hearts from females increased fibrosis on *day 14* pi. Immune and inflammation markers increased in hearts, with some sex differences. These results are the first to identify myocardial stress responses following SARS-CoV-2 infection and reveal sex differences that may contribute to differential outcomes. Future research is required to define the pathways involving these stress signals to fully understand the myocardial effects of COVID-19 and identify targets that mitigate cardiac injury following SARS-CoV-2 infection.

**NEW & NOTEWORTHY** Cardiovascular disease is a leading risk factor for severe COVID-19, and cardiovascular pathologies are among the most common adverse outcomes following SARS-CoV-2 infection. Females and males have different outcomes and adverse cardiovascular events following SARS-CoV-2 infection. This study shows sex differences in stress proteins p62/SQSTM1, ERK1/2, and GSK3α/β, along with innate immunity and inflammation in hearts of ferrets infected with SARS-CoV-2, identifying mechanisms of COVID-19 cardiac injury and cardiac complications of long COVID.

Listen to this article’s corresponding podcast at https://ajpheart.podbean.com/e/cardiac-sex-differences-and-covid/.

## Graphical abstract

**Figure:**
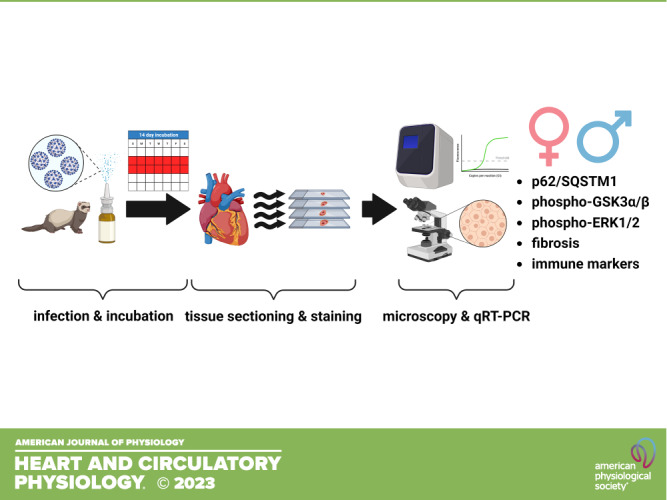

## INTRODUCTION

In late 2019, the novel severe acute respiratory syndrome coronavirus 2 (SARS-CoV-2) emerged from the animal reservoir and into the human population leading to the global coronavirus disease from 2019 (COVID-19) pandemic. With the respiratory system providing the primary route for infection and the devastating impact of SARS-CoV-2 infection on the lungs, COVID-19 was appropriately classified as a respiratory condition. However, cardiovascular involvement was also identified as a critical outcome of SARS-CoV-2 infection early in the pandemic. Importantly, preexisting cardiovascular disease was, and remains, one of the most significant risks for adverse outcomes, including mortality, and a number of cardiovascular pathologies such as blood clots, myocardial infarction, and heart failure have emerged as some of the most common adverse outcomes following SARS-CoV-2 infection (1–3).

It remains unclear the extent to which SARS-CoV-2 can infect cardiac myocytes and if cardiac complications are mediated primarily through direct infection and damage of cardiac myocytes, indirectly through infection of nonmyocyte cells in the heart, or some combination of these mechanisms (4–7). Understanding the molecular basis of cardiovascular complications of COVID-19 is critical to creating therapeutic tools that specifically and precisely target detrimental changes in the heart while leaving intact compensatory responses that may help protect the heart against injury. Despite the widely recognized risk COVID-19 poses to the heart, few studies have investigated the molecular response to SARS-CoV-2 infection. Among the cardiac proteins identified as candidates for involvement in mediating cardiac injury associated with SARS-CoV-2 infection are p62, ERK1/2, and GSK3α/β to prevent toxic accumulation. With infection, p62 acts as an antiviral agent by targeting the viral capsid and marking it for destruction (8), although in vitro studies report that SARS-CoV-2 may hijack autophagy to promote viral replication (9–11). ERK1/2 activation is critical in the myocardial response to pathological stressors including ischemia-reperfusion injury (12), heart failure (13), and myocarditis (14). ERK1/2 has been implicated in promoting the cytokine storm that affects much of the systematic damage of COVID-19 (15). Similar to ERK1/2, GSK3α/β activation is a driving factor behind the damage of several cardiac stressors (16). Some studies show that treatment with the GSK3α/β inhibitor lithium limits SARS-CoV-2 infection in people, possibly by interfering with viral replication (17, 18). The first objective of this study was to identify and quantify alterations in these myocardial proteins known to be involved in the stress response of the heart to determine if they are affected by SARS-CoV-2 infection in vivo. This information is vital for determining the mechanisms of injury in the heart and identifying potential candidates for therapeutic intervention.

The ferret has been useful for modeling respiratory viruses largely because of its natural susceptibility to human viral isolates, as well as anatomical similarities to the human respiratory tract (19). For the study of SARS-CoV-2, ferrets offer advantages over other animal models such as mice, in that they are naturally susceptible to the original Wuhan strain (20–22). In response to SARS-CoV-2, studies have shown mild disease, including elevated body temperature, sneezing, nasal discharge, and decreased activity (21, 23, 24). Live virus infection has been contained to the upper respiratory tract, namely, the nasal turbinates (21, 24). In addition to these tissues, viral RNA has been detected in the tonsil, intestine, and kidney (21, 24). Infection typically results in some mild necrosis of epithelial cells in the nasal turbinates (23, 24). Taken together, the ferret model of SARS-CoV-2 infection is characterized by live viral infection in the nasal turbinates and overall mild clinical disease.

Over time, SARS-CoV-2 has evolved to produce a number of variants, each with distinct characteristics. Although the virus itself continues to change, one trend that has remained consistent is sex differences in adverse outcomes. It is widely established that males have higher rates of severe illness and mortality than age-matched females (25, 26) and that cardiovascular complications are more common in males (26). Work by Francis and colleagues (27) characterized the immune-related host response in the heart and found that infiltration by inflammatory cells may contribute to cardiac injury and mild dysfunction. Although these data provide important information about the possible mechanisms of cardiac injury that can follow recovery from COVID-19, the exclusive use of male hamsters does not allow for a comparison of sex differences. As such, the basis for sex differences in cardiac injury remains unclear and demands a comparative investigation of the cardiac changes driven by SARS-CoV-2 infection in each sex. The second objective of this study was to compare the myocardial stress response of SARS-CoV-2 infection in intact male and female ferrets to identify variations that may be responsible for the differences in cardiovascular outcomes between males and females.

## METHODS

### Animal Ethics

Animal use was approved by the University Animal Care Committee Animal Research Ethics Board from the University of Saskatchewan in association with the Vaccine and Infectious Disease Organization (VIDO-InterVac) (AUP 20200016). All work was conducted in accordance with the Canadian Council of Animal Care and ARRIVE guidelines.

### SARS-CoV-2 Infection

Infection of ferrets was done as previously described (24). The SARS-CoV-2 isolate/Canada/ON/VIDO-01–2020 used for infections was isolated from a patient presenting at a Toronto hospital (28). Adult (1 yr old) female and male ferrets with intact sex organs were purchased from Triple F Farms (Gillett, PA). Ferrets were anesthetized with 5% isoflurane for intranasal infection (10^6^ TCID_50_) in a CL3 facility at VIDO-InterVac. On *days 2*, *7*, and *14* postinfection (pi), ferrets were terminally bled by intracardiac puncture. Uninfected animals were used as controls.

### Tissue Sectioning

Cardiac tissues collected for histopathology and immunohistochemistry were submersed in formalin (8 days at 4°C) and then paraffin-embedded, sectioned, and slide mounted as described previously (24). Slides were transferred to the University of Guelph for staining and imaging.

### Masson’s Trichrome Staining for Interstitial Fibrosis

Mounted tissue samples were stained with Mason’s Trichrome for assessment of interstitial fibrosis. Sections were deparaffinized and rehydrated through several changes of xylene (3 × 2 min) and 100% isopropanol (3 × 2 min), before being incubated once in 70% isopropanol (2 min), and then deionized water (2 min). Rehydrated sections were incubated in Bouin’s solution (Epredia, 57211, Fisher Scientific, Ottawa, ON, Canada) heated to 56°C for 15 min and then rinsed with deionized water. Sections were stained with Weigert’s iron hematoxylin solution [Epredia, mix of equal quantities of parts A (87019) and B (87020)], Fisher Scientific, Ottawa, ON, Canada) for 10 min and rinsed with deionized water, followed by incubation in acid Fuschin solution (F97-25, Fisher Scientific, Ottawa, ON, Canada) for 3 min and another rinse with deionized water. Staining was differentiated by incubating in phosphomolybdic acid solution (MilliporeSigma, 221856, Oakville, ON, Canada) for 1.5 min, rinsing with deionized water, and transferring to light green solution (MilliporeSigma, O3382-25, Oakville, ON, Canada) for 1.5 min. Sections were rinsed with deionized water and dehydrated with successive isopropanol baths of 95% (2 min) and 100% (3 × 2 min) followed by clearing in changes of xylene (3 × 2 min). Samples were coverslipped with Cytoseal (23–244-257, Fisher Scientific, Ottawa, ON, Canada) and left to dry overnight in a fume hood.

### Immunohistochemistry

To determine the expression and localization of cardiac proteins, tissue sections were stained and analyzed by immunohistochemistry (IHC). Sections were deparaffinized and rehydrated to deionized water (as described in *Masson’s Trichrome Staining for Interstitial Fibrosis*). Sections were then quenched in 3% hydrogen peroxide for 20 min at room temperature followed by rinses with phosphate-buffered saline (PBS, 3 × 2 min). Samples underwent antigen retrieval in a citrate buffer solution heated to 95°C for 12 min and were left to cool at room temperature for 20 min. Samples were rinsed three times in PBS (2 min) and then blocked in 5% normal goat serum (NGS; Vector, Burlingame, CA) diluted in PBS for 1 h at room temperature. Primary antibodies were diluted in PBS [phosphorylated GSK3α/β, Cell Signaling Technology, Cat. No. 9331 s, 1:100; p62/SQSTM1 (p62), Cell Signaling Technology, Cat. No. 5114 s, 1:250] or PBS + 0.3% Triton X-100 (phosphorylated ERK1/2, Cell Signaling Technology, Cat. No. 9102 s, 1:100) and applied overnight at 4°C, with one slide per biological sex, per technical run, serving as the omission control (no primary antibody). The following day, after rinsing with PBS (3 × 2 min), sections were incubated with a biotinylated anti-rabbit secondary antibody diluted in PBS (for phosphorylated GSK3α/β, 1:400; p62, 1:200; phosphorylated ERK1/2, 1:100; BA-9200, Vector, Newark, CA) for 1 h at room temperature. Sections were rinsed again in PBS (3 × 2 min) except for phosphorylated ERK1/2, which was rinsed with PBST (0.1% Tween20, 3 × 2 min), then incubated in horseradish peroxidase diluted in PBS (for phosphorylated GSK3α/β and phosphorylated ERK1/2, 1:100; p62, 1:200; 016–030-084, Jackson ImmunoResearch) for 1 h at room temperature. After rinsing in PBS (3 × 2 min), samples were stained with 3,3′-diaminobenzidine (DAB, VECTSK4100, MJS BioLynx, Brockville, ON, Canada) and then rinsed in deionized water twice (2 min). Samples were counterstained in modified Harris hematoxylin (1 min, 23–24567, Fisher Scientific, Ottawa, ON, Canada) and rinsed in deionized water. Sections were differentiated in acid alcohol [mix of isopropanol (HC500) and HCl (A144), Fisher Scientific, Ottawa, ON, Canada], rinsed in deionized water, blued in ammonia water (ammonium hydroxide, A669, Fisher Scientific, Ottawa, ON, Canada), and rinsed again with deionized water. Sections underwent three changes of 100% isopropanol (2 min), were cleared in three changes of xylene (2 min), coverslipped with Cytoseal (3–244-257, Fisher Scientific, Ottawa, ON, Canada), and left to dry overnight. Antibodies were validated in mouse heart tissue through Western blotting and immunohistochemistry before staining was performed. All images were collected using an Olympus FSX100 microscope (Olympus, Tokyo, Japan) with CellSens software (Olympus, Tokyo, Japan) at ×200 magnification for Masson’s trichome, phosphorylated GSK3α/β, and p62, and ×400 magnification for phosphorylated ERK1/2. One sample per biological replicate, per sex, per time point was stained and analyzed for proteins of interest. For each sample analyzed, five nonoverlapping images were taken, representing both cardiomyocyte and noncardiomyocyte populations. Masson’s trichrome images were assessed with ImageJ software (NIH, Bethesda, MD) and immunohistochemistry was analyzed with ImageJ Fiji software (Wayne Rasband and contributors, NIH, Bethesda, MD) using protocols adapted from Crowe and Yue (29). Phosphorylated GSK3α/β and p62 expression was taken as the average pixel intensity, whereas phosphorylated ERK1/2 expression was presented as percentage of phosphorylated ERK1/2-positive cells. Fibrosis was expressed as percentage of total area.

### Immunofluorescence

To determine the presence and localization of cardiac myofibroblasts, tissue sections were stained for α-smooth muscle actin (αSMA) by immunofluorescence (IF). Sections were rehydrated to deionized water (as stated above), rinsed three times in PBS (2 min), and then blocked in 3% NGS diluted in PBS for 1 h at room temperature. αSMA diluted in PBS [αSMA (1A4), Cell Signaling Technology, Cat. No. 48938, 1:400] was applied overnight at 4°C, with one slide per biological sex, per technical run, serving as the omission control, and αSMA^+^ coronary arteries serving as internal positive controls. Sections were then rinsed with PBS (3 × 2 min) before being incubated with the secondary antibody (Alexa Fluor-488-labeled goat anti-mouse, Life Technologies, 1:400) diluted in PBS for 1 h at room temperature. Sections were then rinsed in PBS (3 × 2 min), then 4′,6-diamidino-2-phenylindole (DAPI; Life Technologies D1306, Carlsbad, CA, 1:5,000) diluted in PBS was applied to all tissues for 2 min at room temperature. Finally, tissues were rinsed with PBS three times (2 min each) and slides were coverslipped using Dako fluorescent mounting medium (Dako Canada S3023, Burlington, ON, Canada). All immunofluorescence images were collected using the Axio Imager D1 Microscope (Carl Zeiss Canada, Toronto, ON, Canada) with an AxioCam 305 camera (Carl Zeiss Canada, Toronto, ON, Canada). Each cardiac section was screened for the presence of αSMA^+^ myofibroblasts, and representative images of myocardium and coronary vessels were taken.

### Quantitative Real-Time PCR

Tissue RNA extraction and host quantitative real-time PCR (qRT-PCR) were done as described by Francis et al. (27). Briefly, cardiac RNA was extracted using an RNeasy Mini kit (Qiagen, Toronto, ON, Canada) according to the manufacturer’s instructions. Reactions were done using iScript reverse transcriptase to produce cDNA followed a StepOnePlus Real-Time PCR system using SYBR Green in 96-well plates. All samples were run in triplicate and average values were used for analysis. qRT-PCR primers are listed in Table 1.

**Table 1.** qRT-PCR primers for mediators of innate/adaptive immunity

Mediator	Forward	Reverse
BACT	TGACCGGATGCAGAAGGA	CCGATCCACACCGAGTACTT
CD3	GGCGGTGGCTGCAATC	TCCAGTAATAGACCAGCAGAAGCA
CD4	CCAAAGCTCTTGGCCATTGTG	GCAGAGCCCAAGGAGAAGCA
CD8	GGTCCTTCTACTGTCACTGGTCATC	TTGGCCTGGGACATTTGC
CD45	CTTTGGGCATTTGCCTTTGC	ATGTTGCAGGAACAGTAGAGGA
CD68	CTGCAGAAATGCGAGTGTCG	CGCTGGGACAGGAGAAACTT
CD206	ACAGCTGTTGGACACTAGGC	TTGTCTTGGTTGCAAACCGC
NF-κB	AGGATCGATCAAAGCCTGAA	CCCTCACCAGGTAACAGAGC
ICAM1	TGGACTACGGTGACTGTGGA	CGGACAATCCCTCTGGTCTA
IL-6	AGTGGCTGAAACACGTAACAATTC	ATGGCCCTCAGGCTGAACT
TNF-α	CCAGATGGCCTCCAACTAATCA	GGCTTGTCACTTGGAGTTCGA
IL-1β	GGACTGCAAATTCCAGGACATAA	TTGGTTCACACTAGTTCCGTTGA

### Statistical Analysis

Data were subjected to a one-way ANOVA followed by a post hoc Tukey test using Kaleidograph software (Synergy Software, Reading, PA). Comparisons were made within each sex across time and between males and females at each time point. *n* = 3 hearts were used for each sex and time point. For imaging experiments, five distinct images were collected from tissue sections taken from each heart and analyzed. Values that deviated by more than 2 standard deviation units from the mean were excluded as outliers. *P ≤ 0.05 was taken as significantly different from control and #*P* < 0.05 compared with males at the same time point. All data are presented as means ± SE.

## RESULTS

### Phosphorylated Glycogen Synthase Kinase-3a/b

**Figure 1.**
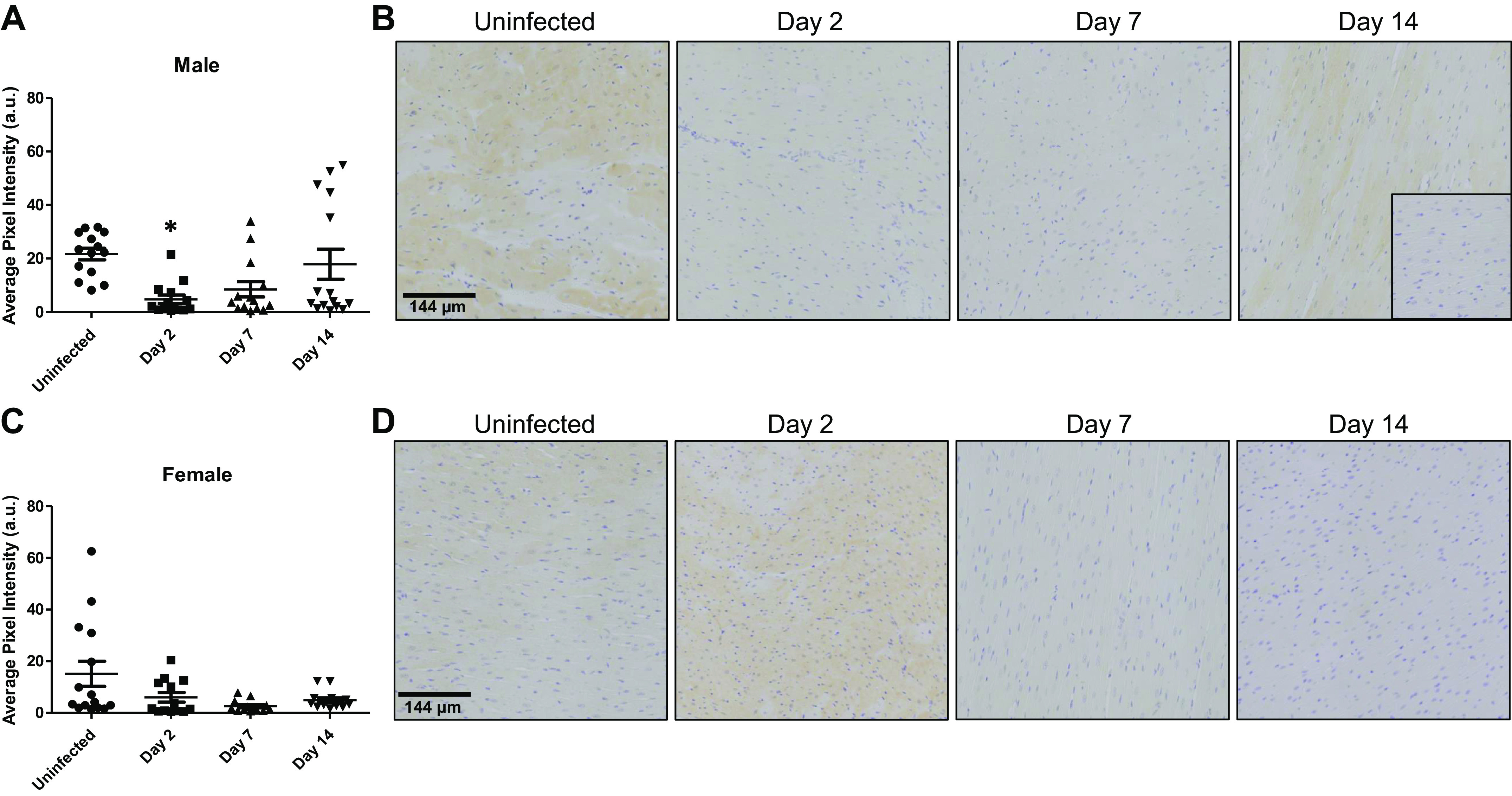
SARS-CoV-2 infection reduces phosphorylated GSK3α/β in the hearts of male ferrets but not in hearts of females. Myocardial sections were probed for phosphorylated GSK3α/β and staining scored semiquantitatively based on the intensity of phosphorylated GSK3α/β-positive cells. *A*: when compared with uninfected control samples, phosphorylated GSK3α/β levels decreased significantly in male ferret hearts on *day 2* postinfection (pi), while changes on *days 7* and *14* pi were insignificant. *B*: representative images of sections from hearts of male ferrets are presented with an insert showing a negative control. *C*: phosphorylated GSK3α/β levels in female control hearts were not significantly affected at any time point up to *day 14* pi. *D*: representative images of sections from female ferret hearts are presented. *n* = 3 hearts in each time point with five sections assessed from each heart. Results are expressed as means ± SE. *P < 0.05 vs. control of same sex. Scale bar = 144 μm.

Phosphorylation of glycogen synthase kinase-3α/β (GSK3α/β) inactivates the intracellular signaling molecule. Myocardial sections taken from male ferrets 2 days pi showed a significant decline in phosphorylated GSK3α/β from 21.7 ± 2.2 arbitrary units (AU) in uninfected controls to 4.8 ± 1.6 AU (*P* = 0.008), representing an increase in activity (Fig. 1, *A* and *B*). By *day 7* pi, phosphorylated GSK3α/β tended to remain lower at 8.5 ± 2.8 AU, but the decrease failed to reach statistical significance (*P* = 0.08). On *day 14* pi, phosphorylation increased to 17.8 ± 5.6 AU, which was not significantly different from baseline levels (*P* = 0.98). Uninfected control samples from the hearts of female ferrets showed no significant differences in phosphorylated GSK3α/β compared with males (*P* = 0.83). At *days 2* (*P* = 0.49), *7* (*P* = 0.12), and *14* (*P* = 0.32) pi, the levels of phosphorylated GSK3α/β were not significantly different from uninfected female ferret hearts, suggesting no effects of SARS-CoV-2 infection on myocardial GSK3α/β activity (Fig. 1, *C* and *D*). Similarly, at no point after infection were phosphorylated GSK3α/β levels different between the sexes (*day 2*, *P* = 1.0; *day* 7, *P* = 0.91; *day 14*, *P* = 0.09). Sections that contained blood vessels showed concentrated staining for phosphorylated GSK3α/β in vascular structures (Supplemental Fig. S1; all Supplemental material is available at https://doi.org/10.5281/zenodo.8206929). There were insufficient sections with blood vessels to quantify staining with the required statistical rigor to determine if phosphorylated GSK3α/β levels changed in the postinfection time frame or if there were sex differences following SARS-CoV-infection.

### p62/SQSTM1

**Figure 2.**
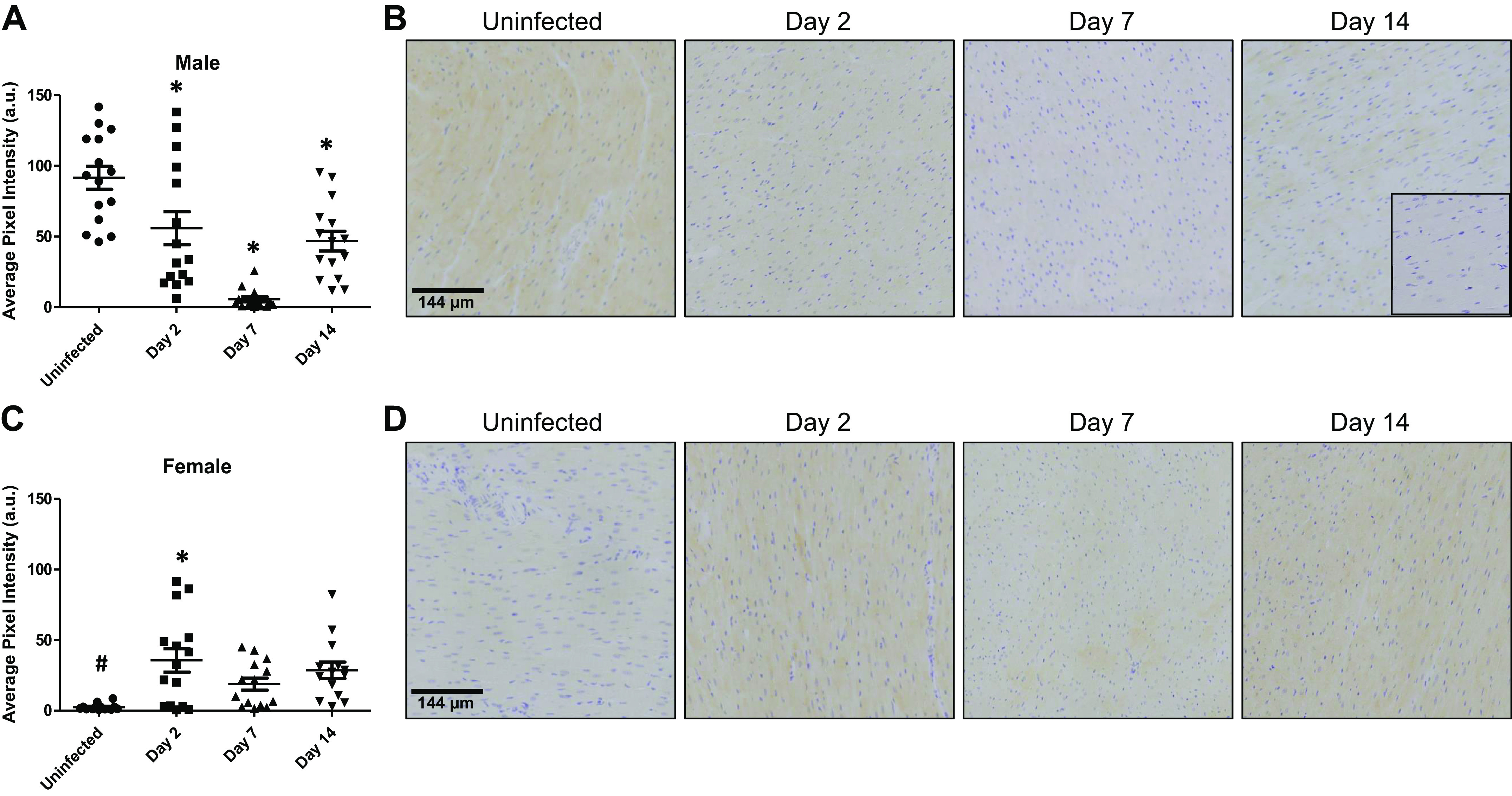
Myocardial p62/SQSTM1 (p62) expression exhibits sex-dependent alterations following SARS-CoV-2 infection. Total p62 expression was quantified in the hearts of male and female ferrets for up 14 days postinfection (pi) with SARS-CoV-2 using immunohistochemistry. *A*: myocardial p62 expression decreased significantly in male ferret hearts at 2 days pi and continued to decline until *day 7* pi. Levels increased at *day 14* pi but remained below uninfected control levels. *B*: representative images of p62 expression in male ferret hearts. *Inset*: negative control. *C*: baseline levels of p62 were significantly lower in female ferret hearts compared with males. p62 levels significantly increased by *day 2* pi in female ferret hearts. *Days 7* and *14* pi levels tended to be above control levels but did not reach statistical significance. *D*: representative images of p62 staining from female ferret hearts. *n* = 3 hearts in each time point with five sections assessed from each heart. Results are expressed as means ± SE. *P < 0.05 vs. control of same sex. #*P* < 0.05 vs. male samples at the same time point. Scale bar = 144 μm.

Baseline p62/SQSTM1 (p62) levels in the hearts of male ferrets were 91.5 ± 8.1 AU, which was significantly higher than the 2.6 ± 0.6 AU measured in the hearts of females (*P* < 0.0001). In the hearts of male ferrets infected with SARS-CoV-2, levels of p62 significantly decreased on *days 2* (*P* = 0.008) and *7* (*P* < 0.0001) pi to 55.9 ± 11.6 AU and 5.6 ± 1.8 AU (Fig. 2, *A* and *B*). p62 expression remained below control levels on *day 14* pi (46.8 ± 7.0 AU, *P* = 0.0002). By contrast, hearts from female ferrets showed significant increases in the levels of myocardial p62 on *day 2* to 35.6 ± 8.3 AU (*P* = 0.02). On *days 7* and *14* pi, p62 levels in the hearts of female ferrets remained above baseline, but the levels of 18.8 ± 4.3 AU (*P* = 0.74) and 28.6 ± 5.8 AU (*P* = 0.17) were not significantly different from baseline. Interestingly, outside of the baseline values, there were no significant differences in p62 levels between the sexes (*day 2* pi, *P* = 0.43; *day 7* pi, *P* = 0.88; *day 14* pi, *P* = 0.59), suggesting that SARS-CoV-2 infection abolished cardiac p62 sex differences. Together these results demonstrate a profound impact of SARS-CoV-2 infection on myocardial p62 levels within the first 14 days of infection and that the effects on p62 differ between the sexes by virtue of their directions of change.

### Extracellular Signal-Regulated Protein Kinase 1/2

**Figure 3.**
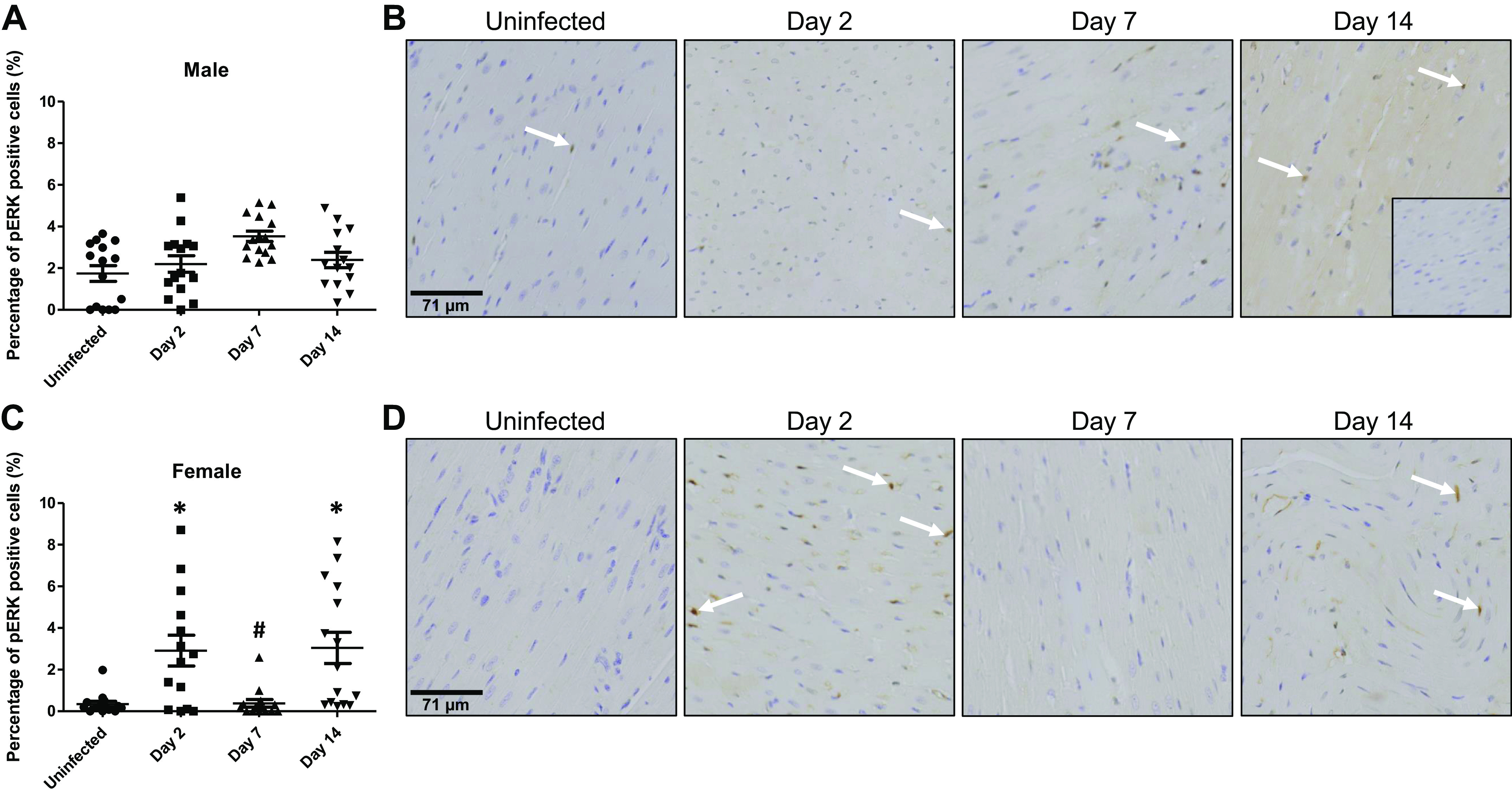
Nuclear localization of phosphorylated ERK1/2 in the heart increases following SARS-CoV-2 infection. Nuclear levels of phosphorylated ERK1/2 were scored semiquantitatively based on counts of phosphorylated ERK1/2-positive cells. *A*: in hearts from male ferrets, the number of cells that positively stained for phosphorylated ERK1/2 was not significantly altered at any time. *B*: representative images showing phosphorylated ERK1/2 in the nuclei of myocardial cells from male ferrets. *Inset*: representative image of a negative control. *C*: nuclear phosphorylated ERK1/2 increased transiently in female ferret hearts on *day 2* pi before returning to baseline levels on *day 7* pi. On *day 14* pi, levels significantly increased above baseline again. On *day 7* pi, the levels of phosphorylated ERK1/2 were significantly lower in female samples compared with those from male ferret hearts at the same time point. *D*: representative images of phosphorylated ERK1/2 in the nuclei of myocardial cells from female ferrets. Positive cells are denoted with white arrows. *n* = 3 hearts in each time point with five sections assessed from each heart. Results are expressed as means ± SE. *P < 0.05 vs. control of same sex. #*P* < 0.05 vs. male samples at the same time point. Scale bar = 144 μm.

In the hearts of male ferrets infected with SARS-CoV-2, levels of activated extracellular signal-regulated protein kinase (ERK) 1/2 (as demonstrated by phosphorylation of the nuclear form) increased from a baseline of 1.7 ± 0.4% of cells to a peak of 3.5 ± 0.3% on *day 7* (*P* = 0.097) and then declined to 2.3 ± 0.4% on *day 14* pi (*P* = 0.97), neither of which was statistically different from baseline (Fig. 3, *A* and *B*). The increase in nuclear ERK1/2 phosphorylation was significant and more rapid in female ferret hearts, increasing from 0.3 ± 0.1% of cells to 2.9 ± 0.7% on *day 2* pi (*P* = 0.004), before declining to 0.4 ± 0.2 at *day 7* (*P* = 1.00) (Fig. 3, *C* and *D*). In heart sections taken from female ferrets, there was a rebound increase in phosphorylated nuclear ERK1/2 on *day 14* to 3.1 ± 0.8% (*P* = 0.001). Although there were no sex differences in baseline levels of phosphorylated nuclear ERK1/2 (*P* = 0.37), levels were significantly lower in female ferret hearts on *day 7* pi compared with males at the same time point (*P* < 0.0001) Overall, our data show SARS-CoV-2 increases nuclear ERK1/2 phosphorylation rapidly and in a biphasic manner in female hearts, whereas males show a tendency toward an increase that peaks on *day 7* pi but does not reach statistical significance at any time point investigated.

### Fibrosis

**Figure 4.**
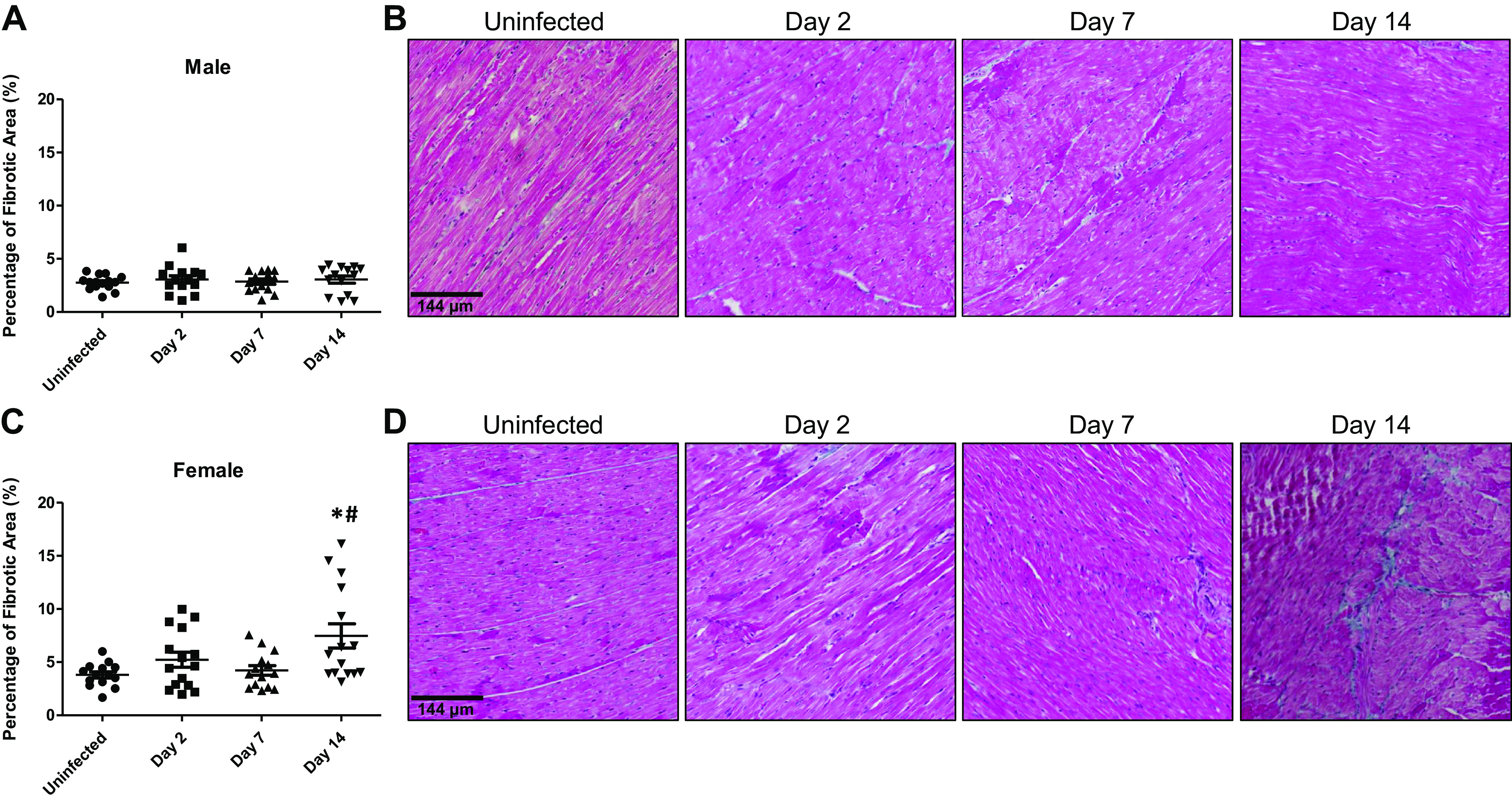
SARS-CoV-2 has a modest impact on interstitial fibrosis in infected ferret hearts. Semiquantification of interstitial fibrosis was done by staining myocardial samples with Masson’s trichrome. *A*: interstitial fibrosis levels in male ferret hearts were unimpacted by SARS-CoV-2 for up to 14 days postinfection (pi). *B*: representative images of male ferret hearts stained with Masson’s trichrome at 2, 7, and 14 days pi. *C*: female ferret hearts exhibited a significant increase in fibrosis 14 days after SARS-CoV-2 infection, but not at earlier time points. *D*: representative images of female ferret heart sections subjected to Masson’s trichrome. *n* = 3 hearts in each time point with five sections assessed from each heart. Results are expressed as means ± SE. *P < 0.05 vs. control of same sex. #*P* < 0.05 vs. male samples at the same time point. Scale bar = 144 μm.

Myocardial injury causing cardiomyocyte death often results in an increase in fibrosis to replace lost cells. Mason’s trichrome staining showed a baseline level of fibrosis of 2.8 ± 0.7% in the hearts of male ferrets that remained unaffected following SARS-CoV-2 infection for up to 14 days (*day 2* pi, *P* = 1.00; *day 7* pi, *P* = 1.00; *day 14* pi, *P* = 1.00; Fig. 4, *A* and *B*). In females infected with SARS-CoV-2, myocardial fibrosis rose from 3.8 ± 1.1% at baseline to 7.5 ± 4.4% on *day 14* pi (*P* = 0.0003) (Fig. 4, *C* and *D*). *Days 2* (*P* = 0.63) and *7* (*P* = 0.99) pi were not significantly different from baseline in female hearts. The increased fibrosis at *day 14* pi in the female ferret hearts was significantly higher than levels seen in male samples at the same time point (*P* < 0.0001). There were no sex differences in the levels of fibrosis before infection (*P* = 0.89). Together these data show a late and modest increase in myocardial fibrosis that is confined to female ferrets infected with SARS-CoV-2.

**Figure 5.**
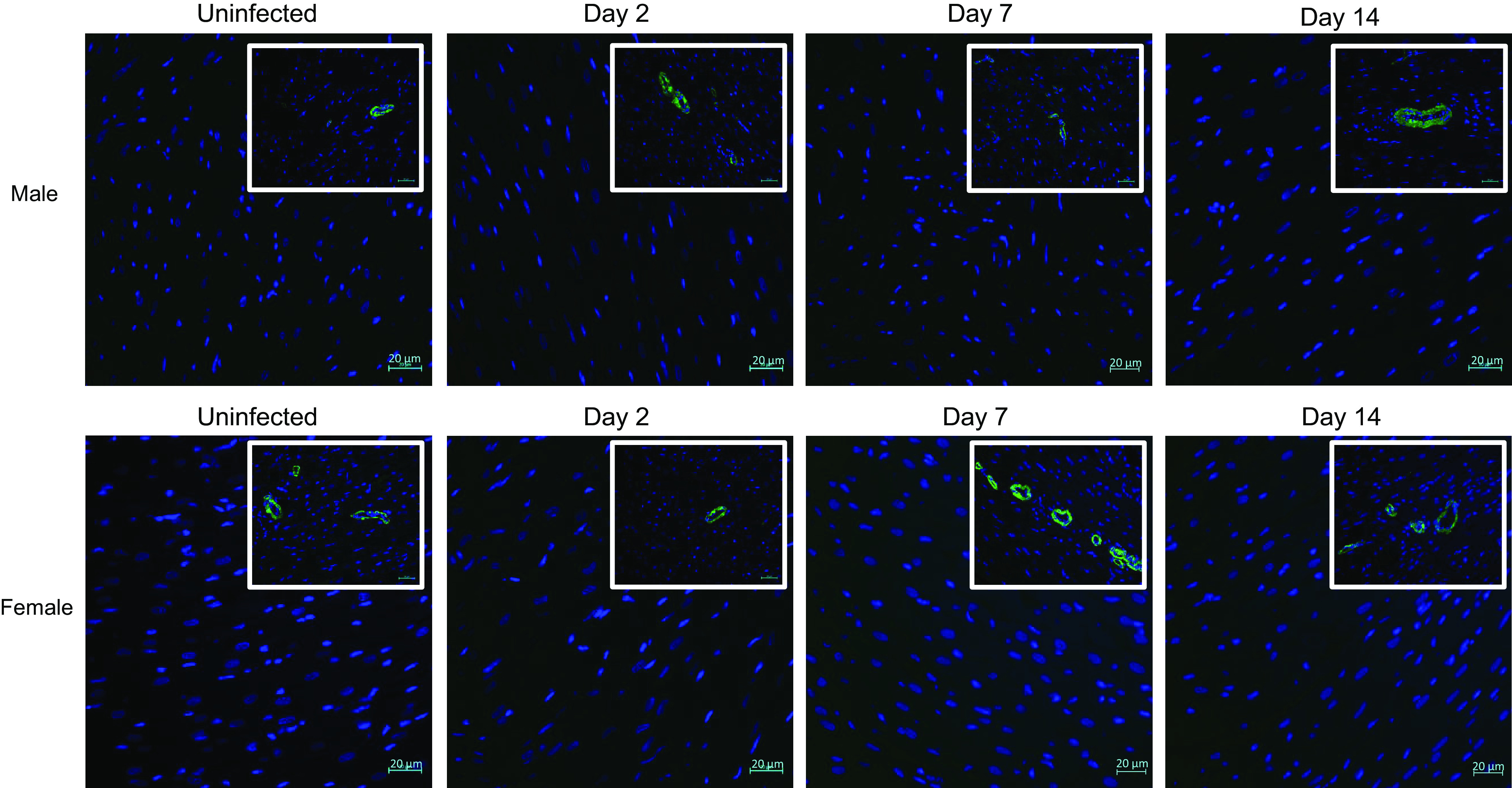
SARS-CoV-2 infection does not increase cardiac myofibroblasts. Myocardial samples were probed with an antibody directed against αSMA to mark myofibroblasts. No myofibroblasts were detected at any time point in either sex. αSMA was detected in coronary arteries (*insets*). *n* = 3 hearts in each time point with five sections assessed from each heart. Scale bar = 20 μm. αSMA, α-smooth muscle actin.

As myofibroblast activation is one of the cellular contributors to cardiac fibrosis, hearts were screened for this cell type by immunostaining for αSMA (Fig. 5). In SARS-CoV-2-infected males and females, no αSMA^+^ myofibroblasts were detected in any heart sections screened. These data suggest that changes in cardiac fibrosis following SARS-CoV-2 infection likely occur independently of myofibroblast activation.

### Cardiac Immunopathology Profiling

**Figure 6.**
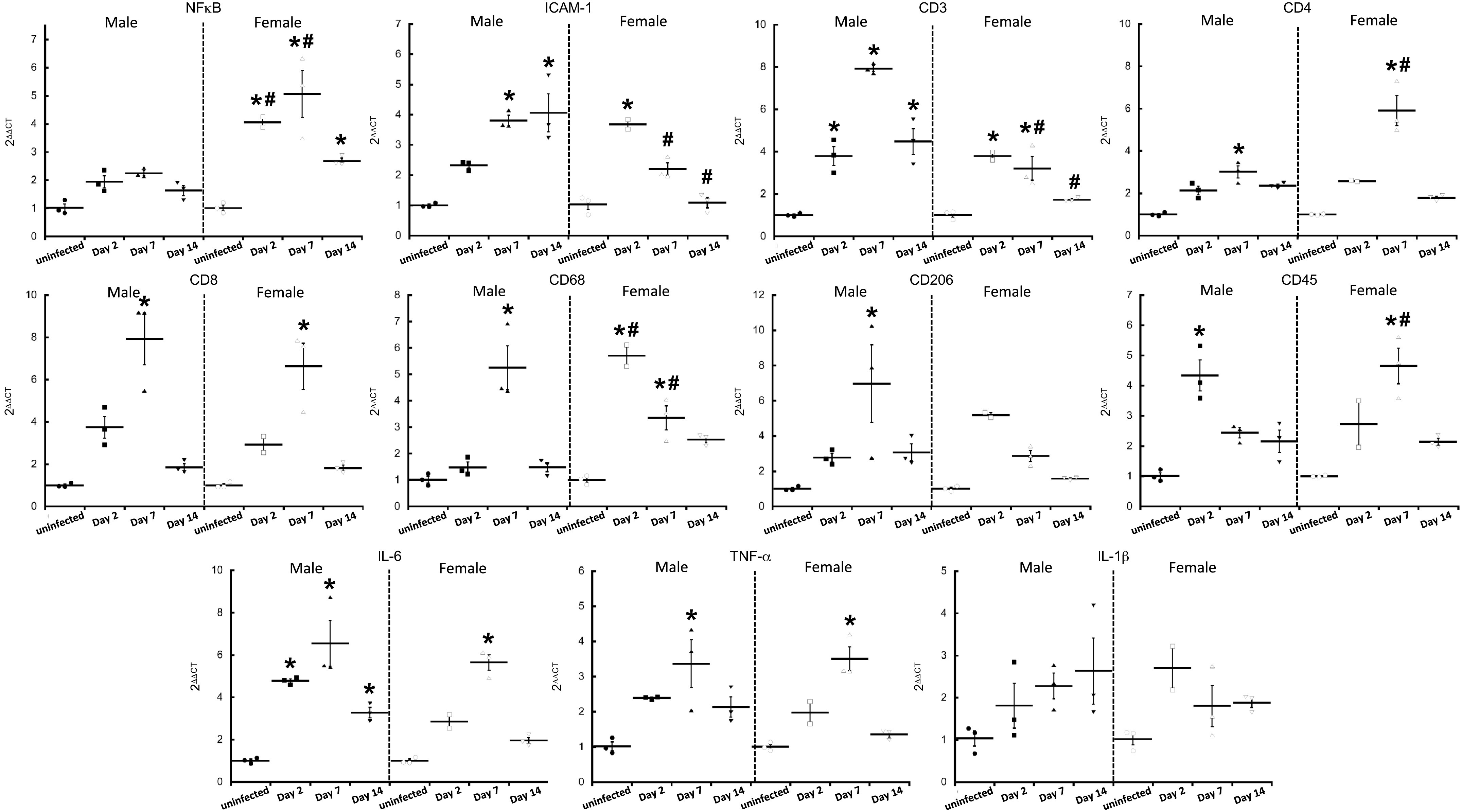
Impact of SARS-CoV-2 on cardiac markers of inflammation and innate immunity. qRT-PCR was performed on RNA extracts from ferret myocardium and assessed for mediators of inflammation and innate/adaptive immunity. Primers used are listed in Table 1. BACT was used as the housekeeping gene and fold changes were calculated using 2^ΔΔCt^ against uninfected controls of the same sex. *n* = 3 hearts in each time point. Results are expressed as means ± SE. *P < 0.05 vs. control of same sex. #*P* < 0.05 vs. male samples at the same time point.

To identify sex differences in the immune response to infection in the heart, we investigated innate/adaptive markers and mediators using qRT-PCR (Fig. 6). The inflammatory marker NF-κB increased above uninfected controls in female ferret hearts on *day 2* pi (*P* = 0.0008) and remained elevated on *days 7* (*P* < 0.0001) and *14* pi (0.047), whereas male hearts showed no significant change at any time point. ICAM-1 levels rose ∼3.6-fold in female hearts by *day* 2 pi (*P* = 0.0003) but showed no significant differences at the other time points, compared with controls. By contrast, ICAM-1 tended to increase on *day 2* pi (*P* = 0.051) in male ferret hearts and reached statistical significance on *days 7* and *14* pi (both *P* < 0.0001). Sex differences in NF-κB were detected on *days 2* (*P* = 0.02) and *7* pi (*P* = 0.0006) when levels were higher in females, whereas ICAM-1 levels were significantly lower in female samples on *days 7* (*P* = 0.01) and *14* pi (*P* < 0.0001).

The T-cell marker CD3 increased ∼3.7-fold in female ferret hearts on *day 2* pi (*P* = 0.003) and remained elevated on *day 7* pi (*P* = 0.009) before decreasing to baseline on *day 14* pi (*P* = 0.83). CD4 and CD8 also increased in female ferret hearts (*P* < 0.0001 and *P* = 0.0003, respectively) on *day 7* while no other time point was significantly affected. Similar patterns in T-cell markers were seen in male ferret hearts where CD3 levels rose on *days 2* (*P* = 0.001) and *7* pi (*P* < 0.001). But unlike female samples that returned to baseline, CD3 levels in the male heart samples remained ∼4.5-fold higher than baseline levels on *day 14* pi (*P* < 0.001). Sex differences in CD3 levels were detected on *days 7* (*P* < 0.0001) and *14* pi (*P* = 0.001) where levels in female hearts were lower than in male hearts. CD4 and CD8 also increased on *day 7* pi (*P* = 0.004 and *P* < 0.0001, respectively) in male ferret hearts, but the increase in CD4 was significantly higher in female samples compared with males (*P* < 0.0001). No sex differences were found in CD8 levels.

The macrophage marker CD68 increased ∼5.6-fold over baseline in female ferret hearts on *day 2* (*P* < 0.0001) and remained significantly elevated on *day 7* pi (*P* = 0.009), before returning to an insignificant (*P* = 0.15) difference on *day 14* pi. In male ferret hearts, the increase in CD68 did not occur until *day 7* pi (*P* < 0.0001), but like the female samples, it was insignificantly changed (*P* = 0.98) on *day 14* pi. Sex differences in CD68 changes were found on *days 2* (*P* < 0.0001) and *7* pi (0.04) when they were higher and lower than male values, respectively. CD206, another macrophage marker, only increased in male ferret hearts and only on *day 7* pi (*P* = 0.003), although this change was not statistically different from levels in female hearts (*P* = 0.06), nor were levels at any other time point.

CD45, a leukocyte marker, increased ∼4.7-fold above baseline (*P* < 0.0001) in female ferret hearts on *day 7* pi, which was significantly higher than male samples at the same time (*P* = 0.01). CD45 levels increased sooner in male hearts, rising to ∼4.3 times the levels of uninfected controls on *day 2* pi (*P* = 0.0002), although this change was not significantly different from female samples taken on the same day pi (*P* = 0.17).

mRNA for inflammatory cytokines IL-6 and TNF-α increased ∼5.6 (*P* < 0.0001) and 3.1 (*P* = 0.0009) times higher than uninfected controls in female ferret hearts on *day 7* pi, while no other time point showed a significant difference for either molecule. By contrast, male ferret hearts exhibited a ∼4.8-fold increase in IL-6 over baseline on *day 2* pi (*P* = 0.0005), which was maintained on *days 7* (*P* < 0.0001) and *14* pi (*P* = 0.04). The increase in TNF-α in male ferret hearts occurred on *day 7* pi (*P* = 0.002), similar to the timing seen in female ferret hearts. IL-1β was not significantly affected following infection in either sex at any time point or at baseline. Despite the apparent differences in timing for IL-6 and TNF-α, no significant sex differences were detected at any time.

## DISCUSSION

The cardiovascular system is a widely recognized and vulnerable target of SARS-CoV-2 infection. Cardiovascular injury as a cause or contributing factor in the deaths of patients with COVID-19 is common and cardiovascular diseases are frequently identified in individuals with long COVID (26, 30, 31). Effectively treating patients to mitigate cardiovascular risks during infection and lower the chances of chronic injury postinfection requires an understanding of how SARS-CoV-2 mediates its effects, including identifying the molecular players in the heart. This study represents the first in vivo investigation of several intracellular messengers that are important to the antiviral response of the heart and drivers of heart dysfunction. We note significant sex differences in the myocardial response by the autophagy protein p62, as well as GSK3α/β and ERK1/2, known mediators of cardiac dysfunction and heart failure. We show that the autophagy protein p62 transiently increased in the hearts of female ferrets infected with SARS-CoV-2, whereas decreased levels are sustained in males throughout the 14-day period of investigation. By contrast, GSK3α/β demonstrated increased activation in male hearts early after infection, but not in females. ERK1/2 activation was achieved rapidly following infection in the hearts of females, along with a second peak on *day 14*, whereas male hearts were unaffected in terms of ERK1/2. Cardiac fibrosis increased in female ferret hearts at *day 14* pi, but this occurred without a concomitant increase in cardiac myofibroblasts. Cardiac inflammation and immune response also demonstrated sex differences during the infection period, in terms of the molecules affected and their timing. In short, stress response elements in the heart were differentially affected by sex, which could contribute to sex-dependent differences in COVID-19 adverse events and outcomes. The identification of these molecular elements and the sex differences that underlie the cardiac response to SARS-CoV-2 infection may be used to develop rational and sex-specific therapies to protect the heart during COVID-19.

### Animal Model

Ferrets represent an excellent preclinical model for a number of infectious diseases, including COVID-19. A previous study by some of us (24) reported the clinical outcomes and host interferon response using the same ferrets that served as donors of the cardiac tissue used in this study. In general, the severity of illness following SARS-CoV-2 infection was described as mild where SARS-CoV-2 viral infection mainly localized to the upper respiratory tract, and no live virus was detected in the lungs or heart. Despite the relatively mild nature of infection, our current results demonstrate a clear impact on the expression and activation of several stress response elements in the heart and do so in a sexually dimorphic manner. These findings may indicate that even with mild or moderate COVID-19, the stress on the heart can create functional changes that are not manifested until after recovery, either in the form of long COVID or cardiac-specific conditions such as heart failure. In fact, early studies examining outcomes 1 year after COVID-19 have already begun to identify an increased risk of cardiovascular complications in people who experienced more mild forms of illness, although the risk appears to increase with the intensity of care (30).

### p62/SQSTM1

p62 is a key component of autophagy, an intracellular recycling system that breaks down proteins, macromolecules, and organelles, and reuses components to maintain cell function and homeostasis. Autophagy is also a mechanism used by cells to destroy pathogens, but some viruses including SARS-CoV and MERS-CoV (32) hijack the autophagy pathway to promote viral replication (33). Some in vitro studies using cultured cells reported that SARS-CoV-2 appears to hijack autophagy through several mechanisms: inhibition of the proton pump ATP6AP1 through the viral NSP6 protein (9), NSP13 binding to host p62 (10), and cleavage of p62 by NSP5 (11), resulting in a decreased viral removal (34, 35). The apparent overlap between viral proteins and their mitigating effects on the innate immune response is not necessarily reflective of redundancy but rather may represent a synergistic system that increases the effectiveness in suppressing immune detection (35). By contrast, Gassen and colleagues (32) found no impact on p62 expression when cultured kidney and lung cells were incubated with SARS-CoV-2, suggesting the impact of SARS-CoV-2 on autophagy is cell specific.

In addition to regulating viral destruction by autophagy, p62 acts as an antiviral factor by stimulating the innate immune response that is important for the acute phase reaction to infection (36). Previous work on Coxsackievirus found a decline in p62 allowed for increased viral replication and resulted in greater heart damage (8). A study by Kim et al. (36) showed p62 activation is critical to protect against the poor outcomes of myocarditis through a mechanism in addition to autophagy.

Our study, which is the first to investigate in vivo p62 changes in the heart following SARS-CoV-2 infection, reveals a significant increase in p62 levels in the hearts of female ferrets shortly after infection. Throughout the COVID-19 pandemic, acute outcomes in females have been better than age-matched males, as measured by hospitalization, intensive care needs, and mortality. A reduction in autophagy and possible hijacking of lysosomes for viral replication represented by increased cardiac p62 levels reported in this study appear to stand in contrast to the more favorable outcomes reported in females. One possible explanation for these results is the dual nature of p62 in the response to infection, specifically its ability to enhance innate immune response to infection. Our data show that increased p62 expression corresponds with a faster and more robust innate immune response in the hearts of female ferrets. Furthermore, ERK1/2, a driver of innate immunity, shows a more rapid myocardial activation in female hearts as compared with males. This innate immunity activation may provide early protection against the infection, but viral hijacking could allow for undetectable levels of the virus to linger, leading to the increased rates of long COVID seen in women.

### GSK3α/β

Females tend to have better outcomes from COVID-19, and our results showing that GSK3α/β activity, as represented by an increase in phosphorylation, is lower in the hearts of female ferrets compared with males infected with SARS-CoV-2, may provide insight into sex-dependent protection. Like other coronaviruses, the SARS-CoV-2 N protein contains a GSK3 phosphorylation site, which is essential for viral transcription and regulation (17, 18). Inhibition of viral replication through a reduction in GSK3α/β activity would allow for reduced tissue injury and faster viral clearance. The faster resolution of inflammatory markers in the hearts of female ferrets suggests a more efficient viral clearance mechanism and is consistent with the sex differences in GSK3 phosphorylation reported here.

Further support for the potentially detrimental role of GSK3α/β in the pathogenesis of COVID-19 is provided through studies demonstrating positive effects in patients treated with the GSK3α/β inhibitor lithium. Lithium directly inhibits GSK3α/β by competing with a magnesium binding site (37). In a US study of 379,611 patients treated with lithium, the rate of COVID-19 infection was half that of the general population (18). Furthermore, therapeutic use of lithium for psychiatric conditions is associated with lower rates of SARS-CoV-2 infection and COVID-19 (38). Although these studies, combined with fundamental research into the role of GSK3α/β in viral replication, support a role for GSK3α/β in COVID-19, some studies report that the toxic levels of lithium required to inhibit coronavirus replication likely limits the potential of this widely used treatment (37, 39). Although lithium toxicity is inconsistently observed in COVID-19 studies and is generally well managed (40), the risk of adverse side effects suggests alternate GSK3α/β inhibitors might be better candidates for prophylactic treatment.

### ERK1/2

ERK1/2 plays a paradoxical role in the response to myocardial stress. On one hand, studies point to the important role of ERK1/2 activation in responding to cardiac stress and helping to drive an adaptive response (12, 41). On the other hand, chronic or uncontrolled activation of ERK1/2 signaling can produce cardiac hypertrophy that increases the risk of cell death and serves a pathological purpose (12, 41).

Even with SARS-CoV-2 infection, ERK1/2 activation has potentially discrepant outcomes. ERK1/2 is necessary for coronavirus RNA synthesis (42) and its activation helps to drive the cytokine storm associated with enhanced COVID-19 pathogenesis and mortality (15). Avolio and colleagues (43) showed that SARS-CoV-2 S protein-mediated damage and apoptosis of pericytes are mediated through an ERK1/2-dependent pathway, providing another link between COVID-19 and myocardial injury. Finally, the SARS-CoV-2 envelope protein binds to Toll-like receptor 2 (TLR2) to increase CXCL8 production, in part through ERK1/2 (15). Early production of chemokines like CXCL8 is critical to enhance T-helper cell function and neutrophil recruitment, but late or sustained increases in CXCL8 are associated with more severe illness and a higher rate of mortality (44).

Our results showing an earlier spike in nuclear phosphorylated ERK1/2 in the hearts of female ferrets is consistent with the more robust and timely immune response to viral infection we also report in this study. Whether ERK1/2 levels in cardiomyocyte nuclei remain at baseline levels or increase to drive cardiac dysfunction observed in some cases of long COVID is not known but represents an important area of focus for future investigations.

Our finding of no change in ERK1/2 phosphorylation in the hearts of male animals following SARS-CoV-2 infection is inconsistent with the work of Cantwell and colleagues (45) who reported increased Akt phosphorylation in male Syrian hamsters infected with SARS-CoV-2. In the RISK signaling cascade, Akt is upstream of ERK1/2 and its activation by phosphorylation increases the phosphorylation of ERK1/2. Besides the different animal models used, one possible explanation for this discrepancy is that the illness produced in hamsters was more severe. Therefore, our work offers unique insight into the more common mild illness associated with SARS-CoV-2 infection and demonstrates for the first time how the response by male and female animals differs.

### Fibrosis

Cardiac myocytes have limited natural regenerative capacity. Their loss through pathological mechanisms is compensated for with the appearance of fibrotic regions in the heart which help to provide structural support but fail to maintain the contractile features. Moreover, fibrosis can interfere with the flow of electrical signals through the heart and increase the risk of cardiac arrhythmias.

Our data show a small growth in fibrotic lesions in the hearts of female ferrets, but not males, 14 days after being infected with SARS-CoV-2. These results are consistent with studies looking at human patients with COVID-19, where cardiac fibrosis was reported in a sizable minority of patients (46). Although no sex-specific studies examining cardiac fibrosis following SARS-CoV-2 infections have been published, the body of work investigating sex differences in pathological remodeling of the heart consistently shows females have lower levels of cardiac fibrosis (47, 48). The results of our study showing an enhanced fibrotic response in females shortly after infection appear to contrast with studies showing a protective effect of sex in females and may represent a unique response to viral infection. In support of this, previous work following infection with the CVB3 virus induced a transient increase in myocardial fibrosis 7 days postinfection that was resolved by 28 days, driven largely by cardiac fibroblasts inducing a temporary proinflammatory chemokine cascade (49).

### Inflammation and Immune Response

Inflammation and a dysregulated immune response have been proposed to cause cardiac injury in cases of severe COVID-19. Determining how these elements manifest in the heart is critical to understanding potential mechanisms of action and developing strategies to mitigate injury. Furthermore, given the well-known sex differences in the immune response to infections, it is imperative to include sex as a variable and not assume that phenomena in one sex are applicable to the other. Our work shows that the ferret model of mild COVID-19 is marked by a rapid T-cell response in the heart that peaks on *day 7* pi and is largely resolved by *day 14* pi. The appearance of macrophage and leukocyte markers in the heart occurs on a slightly faster timeline in females, compared with males. In terms of T cells, cardiac samples from female ferrets infected with SARS-CoV-2 appeared to have a sharper response by virtue of higher peak levels of markers and rapid resolution. Interestingly, inflammatory markers used in this study also displayed marked sex differences: peak NF-κB was higher in females and remained elevated on *day 14* pi, whereas ICAM-1 rose to reach a plateau for the second week after infection. Similarly, the cytokine response showed sex differences with more sustained IL-6 levels in males for up to 14 days pi, which differs from the resolution seen in females during the same period. In general, these markers suggest a more rapid response in the hearts of female ferrets that is largely resolved by *day 14* pi, as opposed to the slightly delayed and sustained inflammation seen in male samples.

A novelty of this study is the inclusion of sex as a variable in the investigation of the cardiac inflammation and immune response to SARS-CoV-2, but others have investigated similar markers in male animals. Viveiros and colleagues (50) used male hamsters and found that CD15, CD68, CD4, and CD8 levels were all elevated on *day 14* pi, whereas the cytokines TNF-α and IL-1β showed some increases on *day 2* that were gone by *day 7*. Francis et al. (27) also used male hamsters to investigate innate/adaptive mediators and found similarly increased levels of CD3 and CD4 in the heart, with transient increases in IL-1β, IL-6, and TNF-α. These findings are in general agreement with our data that showed elevated levels of CD4, CD8, and CD68 in male ferret hearts on *day 7* pi, although we did find a significant increase in TNF-α while Viveiros et al. (50) did not. There is also some discrepancy with respect to IL-1β changes in the heart where Viveiros and colleagues (50) reported an increase of ∼50% that was significantly different from controls on *day 2* pi, and Francis et al. (27) showed a peak on *day 8* pi, whereas we found no significant differences at any time. In addition to the differences in the animal models used across these three studies, Viveiros et al. (50) used both the ancestral and delta variants of SARS-CoV-2, whereas our study and that of Francis et al. (27) only used the original variant for infection. A limited investigation of human heart samples was also done by Viveiros et al., looking at CD68, CD8, and CD4. While only an increase in CD68 was statistically significant, the general trend toward increased levels of these markers agrees with our work and that of the studies using hamsters as disease models. Nonetheless, there appears to be substantial agreement between the ferret and hamster models in terms of cardiac inflammation and immune response.

### Long COVID

Although the focus of this study was the acute response of the heart to SARS-CoV-2 infection, alterations in p62, GSK3α/β, and ERK1/2, along with the innate immune response and cardiac inflammation, may shed some insight into postacute sequelae of COVID-19 or “long COVID.” Long COVID is poorly defined because of the number and variations in reported symptoms, but it is generally characterized as a prolongation or reoccurrence of COVID-19 symptoms 3 mo from diagnosis and lasting for at least 2 mo, without an alternate diagnosis (51). The causes of long COVID are equally uncertain and may be the result of viral persistence, prolonged inflammation, or virus-triggered autoimmunity (52).

Females are more susceptible to long COVID (31) despite having similar rates of infection and better outcomes from acute infection compared with males (25). The reason for the female bias in long COVID is unknown but our observation of increased cardiac p62 levels hints at a possible explanation. Although increased p62 may be indicative of a more robust stimulation of innate immunity, some studies noted that this change is indicative of a blockade of autophagy driven by viral hijacking (32, 35). A study by Stein et al. (53) demonstrated viral persistence in a number of organs, including the heart, in postmortem examinations up to 7 mo after infection. They speculated that the less efficient clearance of virus from nonrespiratory tissues may act as a form of viral seeding but were unable to offer a mechanism for viral persistence in these organ systems. Our data showing increased p62 expression in the hearts of female ferrets after SARS-CoV-2 infection are consistent with diminished viral removal by autophagy and leave open the potential for prolonged viral replication, a hypothesized mechanism for long COVID. In this model, p62 stimulation of innate immunity decreases viral load to low and likely undetectable levels, but the failure of autophagy to fully clear the virus leaves reservoirs of SARS-CoV-2 to produce chronic, low-grade infections and cause the symptoms of long COVID. Future studies should investigate whether autophagy removal of SARS-CoV-2 is impaired in human populations and whether persistent viral load in nonrespiratory tissues is consistently associated with long COVID.

Similarly, the increase in nuclear ERK1/2 phosphorylation could create chronic cardiac issues that cause some symptoms associated with long COVID. Excessive increases in EKR1/2 activity may overstimulate cytokine production to create lasting damage to the heart. In females, we observe an early increase that dissipates by *day 7* pi, but at *day 14* pi, there is a tendency to return toward elevated levels. Whether this trend continues after viral clearance or whether a later surge in cytokine production causes myocardial injury is beyond the timeline of this study. Future studies should investigate the chronic activity levels of these and other cardio-damaging signaling pathways to determine if they are altered in the postinfection state in ways that cause cardiovascular dysfunction.

### Sex Hormones

Sex differences in infectious disease outcomes are not unique to COVID-19 and research generally shows that females have a protective advantage, due in part to relatively higher levels of estrogens than in men (54). Further evidence for a protective role of estrogens in COVID-19 is found in the higher mortality rates in postmenopausal women and mitigation with estrogen supplementation (55, 56). The mechanisms by which estrogens mediate protection against adverse outcomes including cardiac injury have not been established, but several hypotheses have been advanced including a tempered immune response. Gao et al. (57) reported that higher physiological levels of 17β-estradiol in women help mitigate the cytokine storm of COVID-19 by suppressing the expression of the OAS family of genes, offering some level of protection against cardiac injury. In our study, we failed to find the predicted attenuation of IL-1β and TNF-α, although IL-6 tended to resolve faster in females. However, our results are consistent with the work of MacArthur et al. (56) who found that the effects of sex hormones on COVID-19 outcomes are not mediated through cytokine changes in patients who are hospitalized.

The effects of testosterone depend on the stage of COVID-19, with immunosuppressive effects enhancing infection risk, along with an upregulation of TMPRSS2 and enhanced cell entry of SARS-CoV-2 (58). However, once infection is established, testosterone can affect a stronger cytokine response that mediates injury in a number of organ systems, including the heart (59). As noted above, we see some signs of a stronger cytokine response in the hearts of male ferrets, although these patterns are restricted to a tendency toward a faster resolution in females. Interestingly, while testosterone has been proposed as a risk for adverse COVID-19 outcomes, numerous studies report that low testosterone is associated with a higher risk of infection and more severe illness (60), but only in males (56). The impact of low testosterone on cardiac injury in patients with COVID-19 has not been investigated.

### Limitations

The current study investigated key molecular markers that are sentinels for cardiac stress and may correlate with or predict altered cardiac function, but we were unable to measure cardiac performance during the infection period. Previous work by Francis et al. (27) used blood-borne markers in Syrian hamsters infected with SARS-CoV-2 to indirectly measure cardiac function. While only minor changes in potassium and alanine transferase were detected, there was evidence of eosinophilic myocarditis suggesting some level of cardiac dysfunction (27). It should be noted that this work used male Syrian hamsters and the impact of SARS-CoV-2 infection on hearts from female hamsters was not investigated. Future studies dedicated to investigating time- and sex-dependent changes in cardiac function associated with SARS-CoV-2 infection are warranted to determine how males and females differ in their functional risk profiles and to identify key windows for clinical assessment.

Our choice of target proteins is based on a rationale presented earlier in this paper and is limited by a number of factors, including sample and antibody availability. A more complete understanding of the signaling cascades involved in the cardiac injury caused by SARS-CoV-2 infection will require a wider investigation of molecular mediators and confirmation of their activity with complementary assays. This investigation represents an initial foray into these systems and the sex differences that characterize their responses and is necessarily limited in its depth.

Sample size values represent an important limitation of this work, driven in large part by the inability to infect and manage large numbers of ferrets for investigation. While we recognize this limitation, the animal numbers used in this study are not significantly different from other studies that have used animal models to investigate cardiac changes associated with SARS-CoV-2 infection (50, 61).

The heart consists of a number of cell types, and each may respond differently to stressors like viral infection. In an attempt to explain the increase in fibrosis observed in females following SARS-CoV-2 infection, we sought to identify the presence of myofibroblasts as these cells are known mediators of cardiac fibrosis following cardiac injury and during pathology (62). In the ferrets, we did not detect any αSMA^+^ myofibroblasts following SARS-CoV-2 infection, even in the female ferrets at 14 days postinjury in which an increase in fibrosis was observed. These results demonstrate that fibrosis deposition following SARS-CoV-2 infection is not primarily driven through myofibroblast activation, and instead occurs through another cellular mechanism. In an attempt to further determine which cell types might be responsible for the molecular responses reported here, we looked into identifying cardiac fibroblasts. Unfortunately, none of the antibodies tested were able to recognize fibroblast cell markers in ferret hearts, eliminating the opportunity to investigate this cell type in further detail.

The purpose of this study was to investigate the cardiac response to SARS-CoV-2 infection. The 14-day period chosen represents the time required for infection to develop and be cleared in ferrets. As such, the application of our results is limited to the infection period and these findings do not necessarily explain the long-term consequences of COVID-19. Nonetheless, our work may shed some light on the condition commonly known as long COVID by virtue of the fact that some of the observed changes are known to have long-term cardiac consequences and that these molecules can trigger a cascade of events that manifest for weeks and months after the initial stressor. This extension is clearly seen in the development of heart failure when an acute event like myocardial ischemia initiates a series of changes that ultimately manifest as chronic myocardial dysfunction.

### Conclusions

Our results showing potentially pathological alterations in signaling pathways, cardiac remodeling, as well as the innate immune response and inflammation, provide important insights into the basis of cardiac injury associated with COVID-19. It is particularly striking that the myocardial response to SARS-CoV-2 is rapid, even in the face of mild illness. The ability of ERK1/2-, GSK3α/β-, and p62-dependent pathways to mediate both acute and chronic changes in the heart lays the foundation for future investigations to determine how injury sustained during infection creates long-lasting cardiac dysfunction, or what triggers are responsible for the postinfection challenges faced by people with long COVID. Finally, the clear and important differences in myocardial effects of SARS-CoV-2 infection seen between males and females underscore the need to consider sex as a biological variable in COVID-19 management and to create treatment strategies that specifically target the problems that drive adverse cardiovascular outcomes in females and males.

## DATA AVAILABILITY

Data will be made available upon reasonable request.

## SUPPLEMENTAL DATA

10.5281/zenodo.8206929 Supplemental Fig. S1: https://doi.org/10.5281/zenodo.8206929.

## GRANTS

This work was supported with a Heart and Stroke Foundation of Canada Senior Career Investigator Award for Improving the Heart and Brain Health for Women in Canada (to W.G.P.) and Natural Sciences and Engineering Research Council of Canada Discovery Grant 04732 (to W.G.P.). S.R. is supported with funding from Heart and Stroke Foundation Grant-in-Aid G-21–0031543 (to W.G.P.). K.J. is supported with funding from Natural Sciences and Engineering Research Council of Canada Grant 400358. A.A.K. was funded by the Canadian 2019 Novel Coronavirus (COVID-19) Rapid Research Funding Initiative from the Canadian Institutes of Health Research Grants OV5-170349, VRI-172779, and OV2-170357; Atlantic Genome/Genome Canada, Scotiabank COVID-19 IMPACT Grant; the Nova Scotia COVID-19 Health Research Coalition; and Canadian Institutes for Heath Research Operating Grant: Emerging COVID-19 Research Gaps and Priorities for Vaccines and the Canadian Institutes for Heath Research and Coalition for Epidemic Preparedness Leadership Award in Vaccine Research.

## DISCLAIMERS

This article is published with the permission of the Director of VIDO manuscript no. 1019. VIDO receives operational funding from the Government of Saskatchewan through Innovation Saskatchewan and the Ministry of Agriculture and from the Canada Foundation for Innovation through the Major Science Initiatives for its CL3 Facility.

## DISCLOSURES

No conflicts of interest, financial or otherwise, are declared by the authors.

## AUTHOR CONTRIBUTIONS

S.R., K.J., M.E.F., A.A.K., and W.G.P. conceived and designed research; S.R., K.J., M.E.F. and D.F. performed experiments; S.R. and K.J. analyzed data; S.R. and W.G.P. interpreted results of experiments; S.R. and W.G.P. prepared figures; S.R., K.J., M.E.F., and W.G.P. drafted manuscript; S.R., K.J., M.E.F., D.F., A.A.K., and W.G.P. edited and revised manuscript; S.R., K.J., M.E.F., D.F., A.A.K., and W.G.P. approved final version of manuscript.
